# Identification of hypertriglyceridemia based on bone density, body fat mass, and anthropometry in a Korean population

**DOI:** 10.1186/s12872-019-1050-2

**Published:** 2019-03-22

**Authors:** Jeong Hee Chi, Moon Sun Shin, Bum Ju Lee

**Affiliations:** 1 Department of Software, Konkuk University, 120 Neungdong-ro, Gwangjin-gu, Seoul, 05029 Republic of Korea; 2 Department of Computer Engineering, Konkuk University, 268 Chungwon-daero, Chungju-Si, Chungcheongbuk-Do 380-701 Republic of Korea; 3 Future Medicine Division, Korea Institute of Oriental Medicine, 1672 Yuseongdae-ro, Yuseong-gu, Deajeon 305-811 Republic of Korea

**Keywords:** Hypertriglyceridemia, Bone mineral density, Anthropometric characteristics, Triglyceride, Body fat mass, Public health

## Abstract

**Background:**

Hypertriglyceridemia is strongly associated with the risks of cardiovascular disease, coronary heart disease, and metabolic syndrome. The relationship between hypertriglyceridemia or high triglyceride levels and bone mineral density remains controversial. Furthermore, to date, no study has simultaneously examined the association among hypertriglyceridemia, bone area, bone mineral content, bone mineral density, body fat mass, and anthropometrics. The present study aimed to evaluate the association among hypertriglyceridemia, anthropometrics and various bone density and body fat composition variables to identify the best indicator of hypertriglyceridemia in a Korean population.

**Methods:**

The data were obtained from the fifth Korea National Health and Nutrition Examination Survey. In total, 3918 subjects aged 20–80 years participated in this study. In the variable analysis of the waist circumference (WC), trunk fat mass (Trk-Ft), body mass index, etc., a binary logistic regression analysis was performed to examine the significance of the differences between the normal group and hypertriglyceridemia groups.

**Results:**

In both men and women, the WC showed the strongest association with hypertriglyceridemia in the crude analysis (odds ratio (OR) = 1.738 [confidence interval = 1.529–1.976] and OR = 2.075 [1.797–2.397]), but the Trk-Ft was the most strongly associated with the disease after adjusting for age and body mass index (adjusted OR = 1.565 [1.262–1.941] and adjusted OR = 1.730 [1.291–2.319]). In particular, the Pelvis area (Plv-A) was the most significant among the bone variables in women (adjusted OR = 0.641 [0.515–0.796]). In the predictive power analysis, the best indicator of hypertriglyceridemia was WC in women (the area under the receiver operating characteristic curve (AUC) = 0.718 [0.685–0.751]) and Trk-Ft in men (AUC = 0.672 [0.643–0.702]). The WC was also the most predictive among the anthropometric variables in men (AUC = 0.670 [0.641–0.700]). The strength of the association and predictive power was stronger in women than in men.

**Conclusions:**

The WC in women and Trk-Ft in men exhibited the best predictive power for hypertriglyceridemia. Our findings support the use of basic information for the identification of hypertriglyceridemia or high triglyceride levels in initial health screening efforts.

## Background

Hypertriglyceridemia is a well-known vascular risk factor that is strongly correlated with the risks of cardiovascular disease (CVD) [1–4] and coronary heart disease (CHD) [5–7]. High triglyceride (TG) levels are also associated with insulin resistance syndrome and metabolic syndrome, as they represent a vascular risk factor [8]. Numerous studies have reported correlations between TG levels and metabolic syndrome [9–11], insulin resistance syndrome [12–14], and abdominal obesity [9, 12, 15–17].

Hypertriglyceridemia is related to many chronic diseases and is a relatively common disorder; 33.2% of the general population in the 2007 Korean National Survey [18] and 33% of adults in the United States [19] have TG levels above 150 mg/dL. For decades, numerous studies have investigated the best indicators of hypertriglyceridemia, and high TG levels, i.e., hypertriglyceridemia, are strongly associated with anthropometric measures, such as waist circumference (WC) [20–22], the waist-to-hip ratio (WHR) [20], the waist-to-height ratio (WHtR) [23, 24], and the rib-to-forehead circumference ratio (RFcR) [24].

Several recent studies have used dual energy X-ray absorptiometry (DXA) to measure body composition and investigate the association between hypertriglyceridemia and bone mineral density and body fat mass. In particular, the association between body fat distribution and TG levels differs according to ethnicity and race [13, 25]. For example, the relationship between body fat distribution variables and TG levels differed among black, white and Hispanic women [25] and between black and white South African women [13]. Some studies have also reported an association between TG levels and the amount of body fat in the upper body [12, 15, 26], particularly trunk fat [4, 27]. Many studies also report a relationship between hypertriglyceridemia or TG levels and bone mineral density (BMD) [16, 28–34]. TG levels are associated with BMD at the trochanter site [32], lumbar spine [33–35], total femoral region [33, 36], and hip region [16]. However, the relationship between hypertriglyceridemia or high TG levels and BMD remains controversial. Some studies have not reported an association between TG levels and BMD at any skeletal sites [37–41].

Many studies have attempted to identify the best indicators of hypertriglyceridemia, but these studies were based only on partial information, such as anthropometrics, BMD, and body fat mass. Most studies investigating bone density consider only BMD, which is calculated as the ratio of the bone mineral content (BMC) and bone area (BA). An accurate indicator must be identified using measurements based on more detailed variables, such as BMC and BA, which affect BMD at all body sites. The primary hypothesis of this study was that anthropometric measures, body fat mas, and BMD are associated with hypertriglyceridemia or TG levels. In the present study, our objective is to comparatively evaluate anthropometric measures, bone density and body fat mass indices as discriminators of hypertriglyceridemia in Korean adults to identify the best indicator of hypertriglyceridemia. Our study simultaneously examined the association among hypertriglyceridemia, BMD, body fat mass, and anthropometrics. The results of this study may aid in the identification of hypertriglyceridemia in initial health screening efforts and the establishment of a model for more precise identification based on a combination of BMD, anthropometrics, and body fat mass data. To the best of our knowledge, no previous studies have analyzed the associations between detailed bone density and body composition variables measured using DXA with hypertriglyceridemia in Korean adults.

## Methods

### Study population and data source

The data used in this study were obtained from the fifth Korea National Health and Nutrition Examination Survey (KNHANES V-1) conducted in 2010, which is a prospective, cross-sectional, nationally representative survey study conducted by the Korea Centers for Disease Control and Prevention [42]. The KNHANES V-1 was approved by the Korea Ministry of Health and Welfare (2010-02CON-21-C). The Institutional Review Boards of Konkuk University and the Korea Institute of Oriental Medicine also approved the access and analysis of open source data from the KNHANES in the present study with a waiver of documentation of informed consent (IRB No. 7001355–201,802-E-063 and I-1805/003–001).

**Fig. 1:**
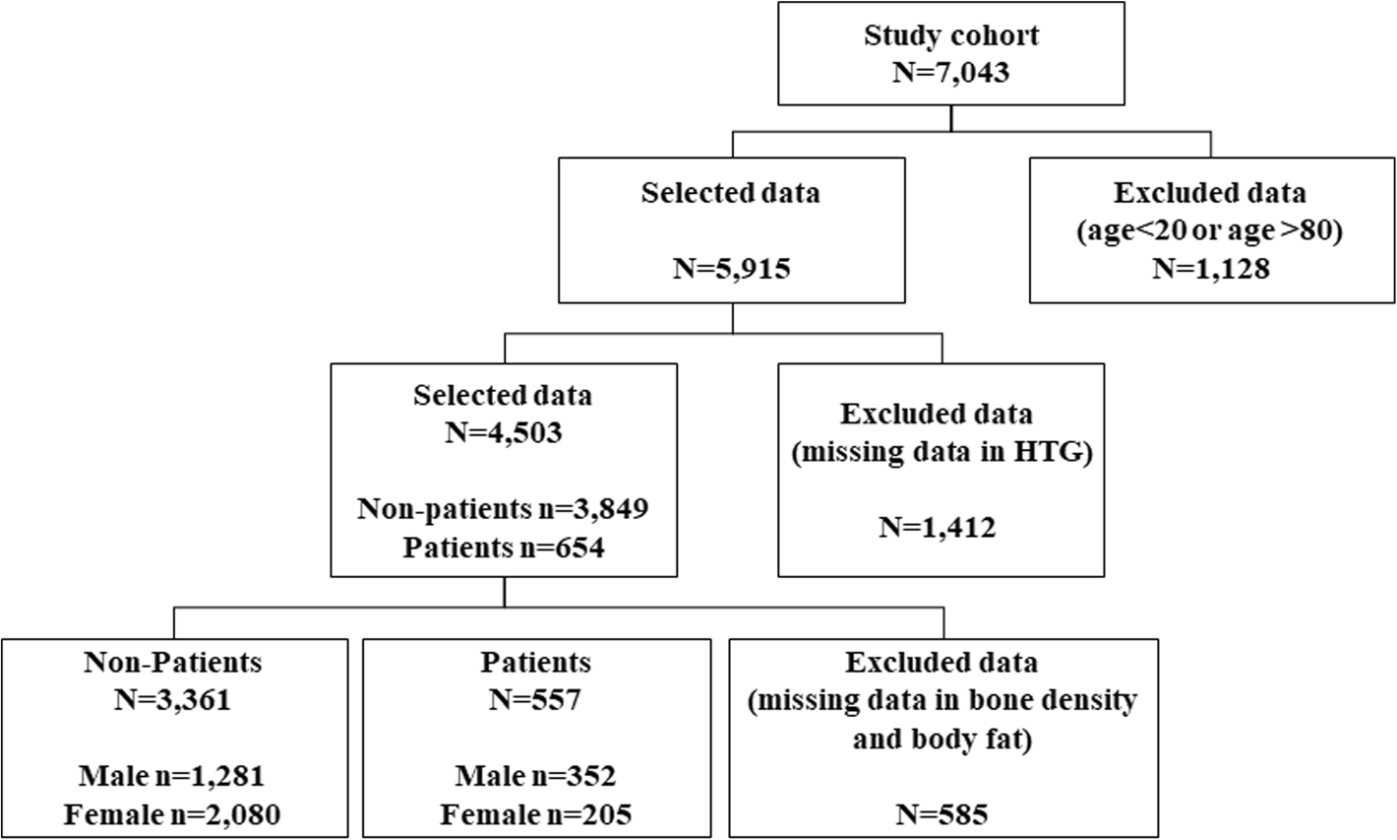
Sample selection procedure. HTG, Hypertriglyceridemia

The KNHANES V-1 included 7043 subjects over the age of 10 years who underwent blood, bone densitometry and body fat composition tests. The National Cholesterol Education Program (NCEP) recommends a measurement of the fasting lipid panel in adults over the age of 20 to evaluate hypertriglyceridemia [43]. We followed the NCEP recommendation. The sample selection procedure included 5915 subjects aged 20–80 years and excluded 1412 subjects who did not fast for 12 h before the health survey. In total, 585 subjects with missing values for the WC, bone density and body fat composition variables were excluded, and data from 3918 subjects were ultimately collected. The final data set consisted of 2285 females (normal: 2080, hypertriglyceridemia: 205) and 1633 males (normal: 1281, hypertriglyceridemia: 352). Figure 1 shows a detailed schematic of the data preprocessing procedure.

### Definition

Hypertriglyceridemia is defined as abnormal TG levels in the blood and is associated with other lipid and metabolic derangements [43]. Hypertriglyceridemia is defined as fasting TG levels ≥200 mg/dL according to the recommendation of the NCEP and previous studies [24, 44, 45]. Therefore, in this study, hypertriglyceridemia was defined as fasting TG levels ≥200 mg/dL.

### Measurement

All anthropometric measurements, such as height, weight, and WC, were recorded using standard methods. Weight was measured with an accuracy of 0.1 kg using an electronic scale (GL-6000-20; Caskorea, Seoul, Korea), and height was measured to the nearest 0.1 cm using a portable stadiometer (Seca 225; Seca, Hamburg, Germany). WC was measured at the midline between the lower rib margin and iliac chest to the nearest 0.1 cm. The body mass index (BMI) was calculated as the weight (kg)/square of height (m2). Blood samples were collected from all participants after a 12-h fast. The total cholesterol (TC), high density lipoprotein cholesterol (HDL-C), low density lipoprotein cholesterol (LDL-C), and triglyceride (TG) levels were analyzed using enzymatic methods (Hitachi Automatic Analyzer 7600, Hitachi, Tokyo, Japan). The bone area, BMC, and BMD of the total femur, trochanter, intertrochanter, femoral neck, ward’s triangle, lumbar spine, left arm, right arm, left rib, right rib, thoracic spin, pelvis, left leg, right leg and whole body excluding the head were measured using DXA (DISCOVERY QDR-4500 W fan-beam densitometer, Hologic, Inc., Bedford, MA, USA). The body fat composition was measured using the same equipment and methods used to measure the BMD. The body fat mass, lean body mass, weight (mass) and body fat percentage were measured in the head, left arm, right arm, trunk, left leg, and right leg.

### Statistical analysis

The statistical analyses were performed using SPSS 21 for Windows (SPSS Inc., Chicago, IL, USA). A binary logistic regression analysis was performed in the crude analyses, and the analyses were adjusted for age and BMI to identify the differences between the normal and hypertriglyceridemia groups after applying standardized transformations to the data sets. Independent two-sample t-tests were performed to statistically assess the gender differences in characteristics. Table 1 provides a detailed description of the demographic characteristics and values of all study variables per group. The area under the receiver operating characteristic curve (AUC) is a major criterion for comparisons of the predictive ability of individual measures.

**Table 1:** Demographic characteristics and values of all study variables in the two groups

Category	Variable	Women	Men	Description
Sample		2285	1633	Number of subjects
Age^*^		48.02 (14.64)	49.23 (15.05)	Age
Anthropometrics	Height (cm)^†^	157 (6.14)	169.8 (6.46)	Height
	Weight (kg)^†^	57.39 (8.68)	69.49 (10.68)	Weight
	WC (cm)^†^	78.00 (9.57)	84.45 (8.82)	Waist circumference
	BMI (kg/m^2^)^†^	23.31 (3.39)	24.06 (3.08)	Body mass index
Bone area (cm^2^)	Wd-A	1.18 (0.10)	1.18 (0.08)	Ward’s triangle area
	LRb-A^†^	128.4 (19.47)	145 (22.23)	Left rib area
	RRb-A^†^	137.4 (24.67)	153.3 (26.83)	Right rib area
	LS-A^†^	53.13 (7.94)	61.26 (8.28)	Lumbar spine area
	Plv-A^†^	171.8 (32.53)	213.5 (32.92)	Pelvis area
Bone mineral content (g)	Wd-BMC^†^	0.67 (0.21)	0.72 (0.21)	Ward’s triangle BMC
	LRb-BMC^†^	79.65 (16.75)	100.8 (20.84)	Left rib BMC
	RRb-BMC^†^	85.88 (18.47)	107.1 (23.55)	Right rib BMC
	LS-BMC^†^	55.12 (14.72)	65.92 (14.91)	Lumbar spine BMC
	Plv-BMC^†^	181.2 (48.61)	242.5 (60.49)	Pelvis BMC
Bone mineral density (g/cm^2^)	Wd-BMD^†^	0.57 (0.16)	0.61 (0.16)	Ward’s triangle BMD
	LRb-BMD^†^	0.62 (0.07)	0.69 (0.08)	Left rib BMD
	RRb-BMD^†^	0.62 (0.07)	0.70 (0.08)	Right rib BMD
	LS-BMD^†^	1.03 (0.17)	1.07 (0.16)	Lumbar spine BMD
	Plv-BMD^†^	1.04 (0.14)	1.12 (0.15)	Pelvis BMD
Fat mass (g)	LArm-Ft^†^	1181 (359.1)	847.4 (299.5)	Left arm fat mass
	RArm-Ft^†^	1193 (367.7)	863.6 (308.4)	Right arm fat mass
	Trk-Ft^†^	10,102 (3450)	9169 (3448)	Trunk fat mass
	WBT-Ft^†^	19,835 (5324)	16,469 (5486)	Whole body total fat mass
Lean mass (g)	LArm-Ln^†^	1711 (274.5)	2952 (486.4)	Left arm lean mass
	RArm-Ln^†^	1822 (290.6)	3090 (495.8)	Right arm lean mass
	Trk-Ln^†^	18,489 (2382)	25,170 (3358)	Trunk lean mass
	WBT-Ln^†^	37,204 (4616)	52,528 (6827)	Whole body total lean mass
Mass (g)	LArm-Ms^†^	2893 (522.8)	3800 (639.5)	Left arm mass
	RArm-Ms^†^	3015 (536.7)	3954 (646.9)	Right arm mass
	Trk-Ms^†^	28,592 (5044)	34,339 (5853)	Trunk mass
	WBT-Ms^†^	57,040 (8565)	68,998 (10534)	Whole body total mass

These results reveal significant differences between men and women by independent two-samples t-tests [means (standard deviations)]. Ward’s triangle is the space formed at the femoral neck by the intersection of three trabecular bundles, namely, the principal compressive, the secondary compressive, and the tensile trabecular [53]

*SD* standard deviation, *BMC* bone mineral content, *BMD*,bone mineral density

^*^ *p* < 0.05; ^†^ *p* < 0.001

## Results

### Associations among hypertriglyceridemia, bone density and body fat mass

Tables 2 and 3 list the associations between hypertriglyceridemia and the anthropometric, bone density and body fat composition measurements in women and men. Among all variables examined in this study, WC displayed the strongest association with hypertriglyceridemia among women in the crude analysis (odds ratio (OR) = 2.075 [confidence interval = 1.797–2.397]), and the association remained highly significant after adjusting for age and BMI (adjusted OR = 1.615 [1.202–2.171]). The trunk fat mass (Trk-Ft) was highly associated with hypertriglyceridemia in the crude analysis (OR = 1.940 [1.691–1.226]) and remained the variable most strongly associated with hypertriglyceridemia after adjusting for confounders (adjusted OR = 1.730 [1.291–2.319]). Of the bone density variables, the pelvis area (Plv-A) displayed the greatest negative association with hypertriglyceridemia in both the crude (OR = 0.487 [0.415–0.571]) and adjusted analyses (adjusted OR = 0.641 (0.515–.796)). Among the body fat variables, Trk-Ft displayed the most significant association with hypertriglyceridemia in both the crude and adjusted analyses.

**Table 2:** Associations between hypertriglyceridemia and bone density and body fat mass in women

Variable	Mean (SD)		Crude		Adjusted	
	Normal group	Patient group	p	OR	p	OR
Age	47.17 (14.53)	56.59 (12.89)	< 0.001	1.957 (1.676–2.285)	–	–
Height	157.2 (6.098)	154.8 (6.158)	< 0.001	0.676 (0.585–0.780)	0.716	0.968 (0.812–1.154)
Weight	57.05 (8.528)	60.76 (9.442)	< 0.001	1.467 (1.288–1.672)	0.951	1.010 (0.736–1.386)
WC	77.35 (9.391)	84.61 (8.807)	< 0.001	2.075 (1.797–2.397)	0.001	1.615 (1.202–2.171)
BMI	23.11 (3.337)	25.32 (3.273)	< 0.001	1.777 (1.558–2.027)	–	–
Wd-A	1.179 (0.103)	1.175 (0.094)	0.548	0.957 (0.829–1.105)	0.345	1.083 (0.918–1.277)
LRb-A	127.7 (19.07)	135.6 (22.02)	< 0.001	1.473 (1.281–1.694)	0.008	1.272 (1.065–1.520)
RRb-A	136.2 (24.40)	149.4 (24.27)	< 0.001	1.684 (1.460–1.942)	0.098	1.172 (0.971–1.415)
LS-A	53.25 (7.868)	51.93 (8.587)	0.024	0.846 (0.731–0.978)	0.253	1.097 (0.936–1.286)
Plv-A	173.7 (32.39)	152.2 (27.05)	< 0.001	0.487 (0.415–0.571)	< 0.001	0.641 (0.515–0.796)
Wd-BMC	0.680 (0.212)	0.603 (0.210)	< 0.001	0.677 (0.579–0.791)	0.678	1.047 (0.844–1.298)
LRb-BMC	79.24 (16.37)	83.76 (19.77)	< 0.001	1.293 (1.127–1.482)	0.007	1.291 (1.071–1.556)
RRb-BMC	85.23 (18.25)	92.38 (19.48)	< 0.001	1.448 (1.261–1.662)	0.128	1.158 (0.959–1.399)
LS-BMC	55.48 (14.65)	51.41 (15.00)	< 0.001	0.746 (0.641–0.869)	0.948	1.006 (0.845–1.198)
Plv-BMC	183.1 (48.14)	161.3 (49.06)	< 0.001	0.620 (0.531–0.723)	0.069	0.827 (0.674–1.015)
LRb-BMD	0.574 (0.160)	0.512 (0.166)	0.362	0.935 (0.809–1.080)	0.468	1.067 (0.896–1.270)
Wd-BMD	0.618 (0.069)	0.613 (0.075)	< 0.001	0.671 (0.577–0.780)	0.784	1.031 (0.829–1.282)
RRb-BMD	0.625 (0.069)	0.617 (0.077)	0.126	0.892 (0.771–1.032)	0.792	1.023 (0.862–1.215)
LS-BMD	1.031 (0.174)	0.977 (0.179)	< 0.001	0.717 (0.615–0.836)	0.291	0.910 (0.764–1.084)
Plv-BMD	1.04 (0.139)	1.040 (0.158)	0.729	0.975 (0.844–1.126)	0.187	1.119 (0.947–1.322)
LArm-Ft	1167 (356.2)	1327 (357.1)	< 0.001	1.487 (1.305–1.694)	0.642	0.947 (0.751–1.193)
RArm-Ft	1177 (364.5)	1349 (364.2)	< 0.001	1.522 (1.335–1.736)	0.841	0.976 (0.772–1.235)
Trk-Ft	9880 (3408)	12,365(3049)	< 0.001	1.940 (1.691–2.226)	< 0.001	1.730 (1.291–2.319)
WBT-Ft	19,601 (5306)	22,208 (4935)	< 0.001	1.560 (1.367–1.782)	0.772	0.958 (0.717–1.280)
LArm-Ln	1705 (268.4)	1771 (325.1)	0.001	1.254 (1.095–1.437)	0.951	1.005 (0.857–1.179)
RArm-Ln	1815 (285.0)	1895 (335.1)	< 0.001	1.293 (1.130–1.479)	0.271	1.093 (0.933–1.280)
Trk-Ln	18,430 (2325)	19,098 (2833)	< 0.001	1.300 (1.136–1.488)	0.766	1.030 (0.849–1.249)
WBT-Ln	37,112 (4513)	38,144 (5484)	0.002	1.236 (1.078–1.416)	0.835	1.021 (0.840–1.242)
LArm-Ms	2873 (511.0)	3098 (594.1)	< 0.001	1.459 (1.284–1.658)	0.792	0.970 (0.776–1.213)
RArm-Ms	2993 (525.8)	3244 (591.1)	< 0.001	1.507 (1.326–1.714)	0.472	1.087 (0.866–1.365)
Trk-Ms	28,309 (4935)	31,464 (5248)	< 0.001	1.740 (1.526–1.985)	0.003	1.594 (1.166–2.181)
WBT-Ms	56,714 (8426)	60,352 (9262)	< 0.001	1.465 (1.285–1.669)	0.987	0.997 (0.727–1.368)

The results of the crude analysis and analyses adjusted for age and BMI were obtained using a binary logistic regression

*SD* Standard deviation, *OR* Odds ratio, *WC* Waist circumference, *BMI* Body mass index, *Wd-A* Ward’s triangle area, *LRb-A* Left rib area,*RRb-A* Right rib area, *LS-A* Lumbar spine area, *Plv-A* Pelvis area, *Wd-BMC* Ward’s triangle bone mineral content, *LRb-BMC* Left rib bone mineral content, *RRb-BMC* Right rib bone mineral content, *LS-BMC* Lumbar spine bone mineral content, *Plv-BMC* Pelvis bone mineral content, *LRb-BMD* Ward’s triangle bone mineral density, *Wd-BMD* Left rib bone mineral density, *RRb-BMD* Right rib bone mineral density, *LS-BMD* Lumbar spine bone mineral density, *Plv-BMD* Pelvis bone mineral density, *LArm-Ft* Left arm fat mass, *RArm-Ft* Right arm fat mass, *Trk-Ft* Trunk fat mass, *WBT-Ft* Whole body total fat mass, *LArm-Ln* Left arm lean mass, *RArm-Ln* Right arm lean mass, *Trk-Ln* Trunk lean mass, *WBT-Ln* Whole body total lean mass, *LArm-Ms* Left arm mass, *RArm-Ms* Right arm mass, *Trk-Ms* Trunk mass, *WBT-Ms* Whole body total mass

**Table 3:** Associations between hypertriglyceridemia and bone density and body fat mass in men

Variable	Mean (SD)		Crude		Adjusted	
	Normal group	Patient group	p	OR	p	OR
Age	49.04 (15.49)	49.89 (13.32)	0.347	1.058 (0.941–1.191)	–	–
Height	167.0 (6.450)	169.2 (6.473)	0.042	0.884 (0.786–0.996)	0.055	0.873 (0.760–1.003)
Weight	68.68 (10.63)	72.43 (10.39)	< 0.001	1.411 (1.255–1.587)	0.084	0.788 (0.602–1.033)
WC	83.43 (8.954)	88.14 (7.212)	< 0.001	1.738 (1.529–1.976)	0.001	1.482 (1.171–1.877)
BMI	23.73 (3.090)	25.24 (2.757)	< 0.001	1.629 (1.443–1.839)	–	–
Wd-A	1.175 (0.085)	1.183 (0.084)	0.119	1.098 (0.976–1.234)	0.165	1.090 (0.965–1.230)
LRb-A	142.9 (21.90)	152.8 (21.72)	< 0.001	1.566 (1.388–1.767)	< 0.001	1.332 (1.144–1.550)
RRb-A	150.9 (26.28)	161.9 (27.08)	< 0.001	1.501 (1.332–1.690)	0.011	1.217 (1.046–1.416)
LS-A	61.22 (8.233)	61.42 (8.447)	0.692	1.024 (0.910–1.152)	0.965	1.003 (0.888–1.133)
Plv-A	215.8 (32.97)	205.1 (31.39)	< 0.001	0.714 (0.632–0.808)	< 0.001	0.661 (0.573–0.763)
Wd-BMC	0.718 (0.211)	0.715 (0.193)	0.823	0.987 (0.877–1.111)	0.299	0.920 (0.787–1.076)
LRb-BMC	99.24 (20.82)	106.3 (20.00)	< 0.001	1.390 (1.237–1.563)	0.322	1.082 (0.926–1.264)
RRb-BMC	105.3 (23.10)	113.6 (24.06)	< 0.001	1.411 (1.255–1.587)	0.365	1.076 (0.918–1.261)
LS-BMC	65.91 (15.07)	65.97 (14.33)	0.950	1.004 (0.892–1.129)	0.272	0.933 (0.825–1.056)
Plv-BMC	243.6 (61.02)	238.2 (58.38)	0.132	0.912 (0.809–1.028)	0.001	0.792 (0.687–0.913)
LRb-BMD	0.609 (0.161)	0.603 (0.148)	0.545	0.964 (0.856–1.085)	0.114	0.877 (0.746–1.032)
Wd-BMD	0.692 (0.081)	0.694 (0.071)	0.768	1.018 (0.905–1.144)	0.011	0.838 (0.731–0.960)
RRb-BMD	0.696 (0.077)	0.699 (0.073)	0.430	1.048 (0.932–1.179)	0.050	0.878 (0.771–1.000)
LS-BMD	1.071 (0.167)	1.069 (0.151)	0.830	0.987 (0.877–1.111)	0.099	0.901 (0.797–1.020)
Plv-BMD	1.117 (0.154)	1.150 (0.153)	< 0.001	1.228 (1.094–1.379)	0.559	1.041 (0.910–1.191)
LArm-Ft	820.2 (303.0)	946.3 (264.2)	< 0.001	1.502 (1.336–1.689)	0.162	1.139 (0.949–1.368)
RArm-Ft	837.3 (311.1)	959.3(278.7)	< 0.001	1.466 (1.304–1.648)	0.524	1.061 (0.884–1.274)
Trk-Ft	8759 (3452)	10,662 (3000)	< 0.001	1.729 (1.530–1.953)	< 0.001	1.565 (1.262–1.941)
WBT-Ft	15,920 (5522)	18,469 (4865)	< 0.001	1.576 (1.399–1.776)	0.052	1.229 (0.998–1.512)
LArm-Ln	2942 (480.6)	2991 (505.7)	0.100	1.103 (0.981–1.239)	0.034	0.850 (0.732–0.988)
RArm-Ln	3083 (489.2)	3118 (519.1)	0.239	1.073 (0.954–1.206)	0.012	0.823 (0.708–0.958)
Trk-Ln	25,010 (3301)	25,754 (3506)	< 0.001	1.244 (1.107–1.398)	0.022	0.814 (0.683–0.971)
WBT-Ln	52,277 (6721)	53,444 (7135)	0.005	1.185 (1.054–1.332)	0.003	0.756 (0.630–0.907)
LArm-Ms	3763 (634.5)	3937 (639.9)	< 0.001	1.304 (1.161–1.464)	0.156	0.869 (0.716–1.055)
RArm-Ms	3920 (642.0)	4078 (650.5)	< 0.001	1.269 (1.129–1.425)	0.027	0.800 (0.657–0.975)
Trk-Ms	33,770 (5803)	36,416 (5567)	< 0.001	1.559 (1.384–1.755)	0.321	1.144 (0.877–1.491)
WBT-Ms	68,197 (10473)	71,913 (10253)	< 0.001	1.414 (1.257–1.590)	0.106	0.801 (0.613–1.048)

The results of the crude analysis and analyses adjusted for age and BMI were obtained using a binary logistic regression

*SD* Standard deviation,*OR* Odds ratio, *WC* Waist circumference, *BMI* Body mass index, *Wd-A* Ward’s triangle area, *LRb-A* Left rib area, *RRb-A* Right rib area, *LS-A* Lumbar spine area, *Plv-A* Pelvis area, *Wd-BMC* Ward’s triangle bone mineral content, *LRb-BMC* Left rib bone mineral content, *RRb-BMC* Right rib bone mineral content, *LS-BMC* Lumbar spine bone mineral content, *Plv-BMC* Pelvis bone mineral content, *LRb-BMD* Ward’s triangle bone mineral density, *Wd-BMD* Left rib bone mineral density, *RRb-BMD* Right rib bone mineral density, *LS-BMD* Lumbar spine bone mineral density, *Plv-BMD* Pelvis bone mineral density, *LArm-Ft* Left arm fat mass, *RArm-Ft* Right arm fat mass, *Trk-Ft* Trunk fat mass, *WBT-Ft* Whole body total fat mass, *LArm-Ln* Left arm lean mass, *RArm-Ln* Right arm lean mass, *Trk-Ln* Trunk lean mass, *WBT-Ln* Whole body total lean mass, *LArm-Ms* Left arm mass, *RArm-Ms* Right arm mass, *Trk-Ms* Trunk mass, *WBT-Ms* Whole body total mass

In men, among all variables, WC exhibited the strongest association with hypertriglyceridemia in the crude analysis (OR = 1.738 [1.529–1.976]), but after adjusting for age and BMI, Trk-Ft exhibited the strongest association with hypertriglyceridemia (adjusted OR = 1.565 [1.262–1.941]). Of the bone density variables, the left rib area (LRb-A) displayed the strongest association with hypertriglyceridemia (OR = 1.566 [1.388–1.767]) in the crude analysis, and this association remained highly significant after adjusting for confounders (OR = 1.332 [1.144–1.550]). Plv-A displayed a strong negative association with hypertriglyceridemia (adjusted OR = 0.661[0.573–0.763]). In the present study, Trk-Ft exhibited the strongest associations with hypertriglyceridemia in both men (OR = 1.565 [1.262–1.941]) and women (OR = 1.730 [1.291–2.319]) in the adjusted analysis.

### Power of bone density and body fat mass in the identification of hypertriglyceridemia

**Fig. 2:**
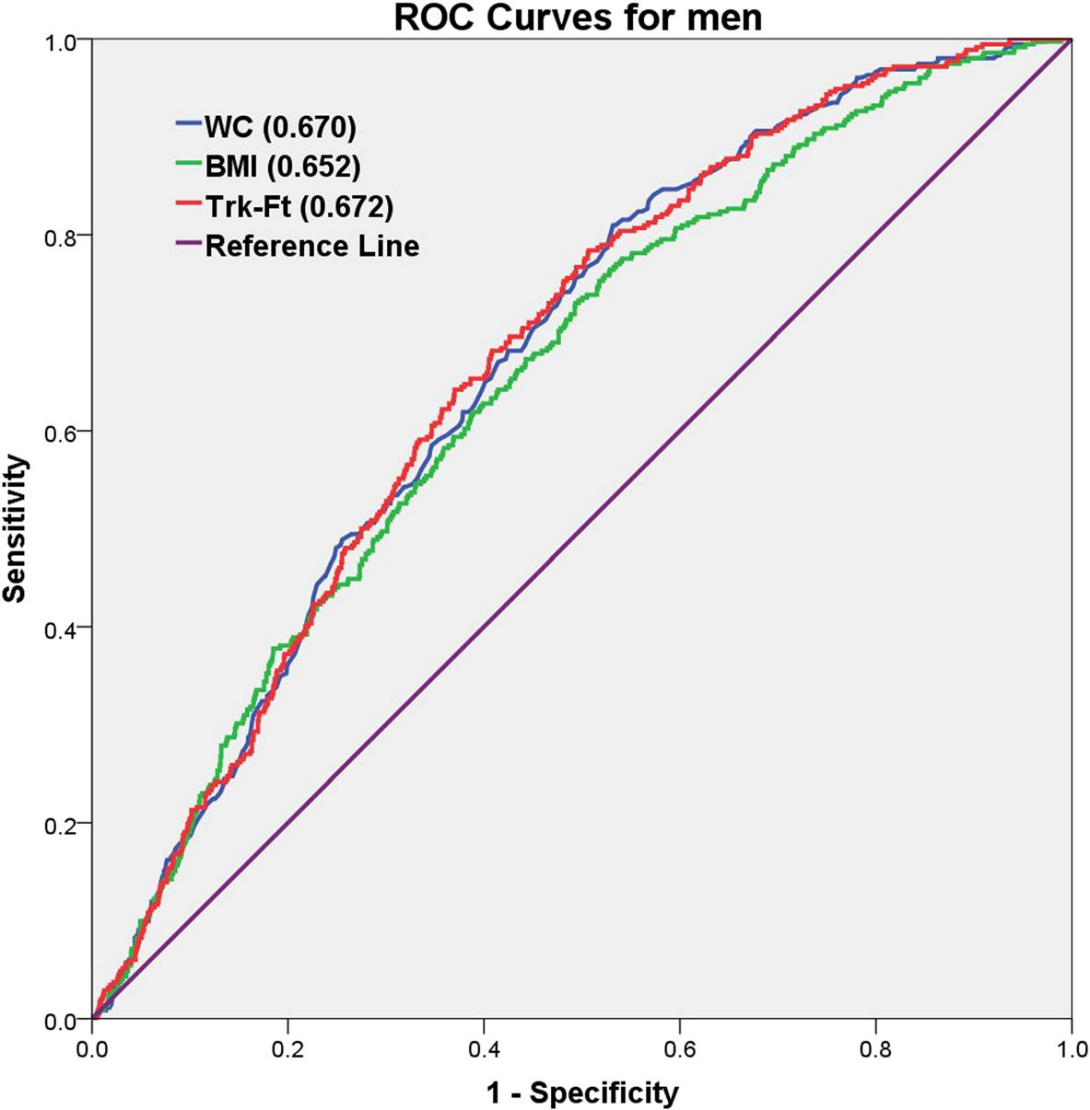
Comparison of the predictive power based on AUCs (the area under a receiver operating characteristic curve) among men. WC, Waist circumference; BMI, Body mass index; Trk-Ft, Trunk fat mass

**Fig. 3:**
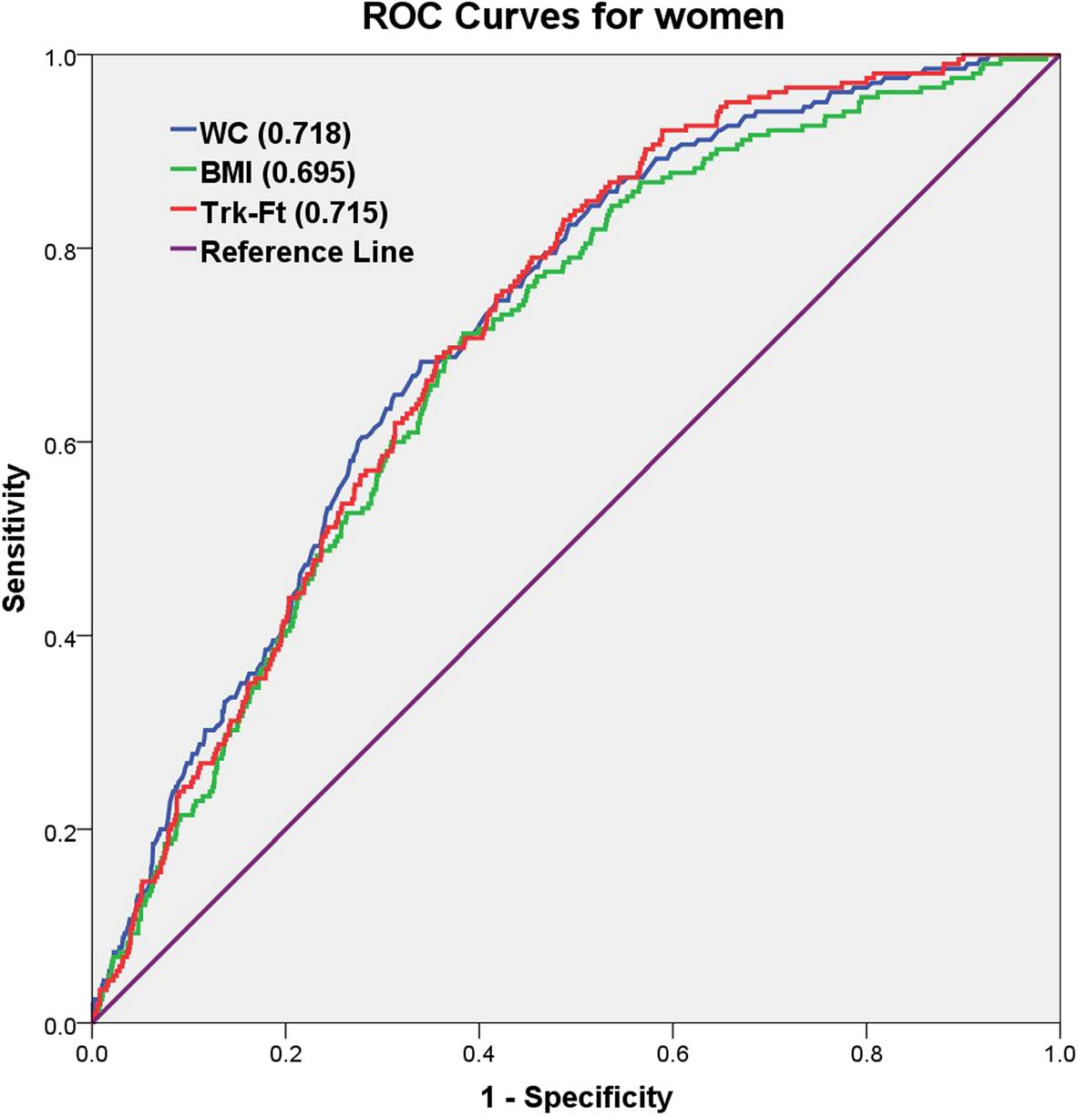
Comparison of the predictive power based on AUCs (the area under a receiver operating characteristic curve) among women. WC, Waist circumference; BMI, Body mass index; Trk-Ft, Trunk fat mass

Table 4 lists the predictive power of all variables in identifying hypertriglyceridemia. WC exhibited the highest AUC value (AUC = 0.718 [0.685–0.751]) in women. Among the bone density variables, Plv-A exhibited a strong predictive power (AUC = 0.696 [0.660–0.731]), and among the body fat variables, Trk-Ft exhibited substantial predictive power (AUC = 0.715 [0.684–0.747]). In men, Trk-Ft exhibited the highest AUC value among all body fat variables (AUC = 0.672 [0.643–0.702]). Of the bone density variables, LRb-A exhibited a strong predictive power (AUC = 0.633 [0.601–0.665]). These results clearly revealed gender differences. Among all variables, WC was the highest overall indicator of hypertriglyceridemia in women, and Trk-Ft was the highest overall indicator in men. The bone density variable Plv-A exhibited the strongest predictive power in women, and LRb-A was the strongest indicator in men. The predictive power of these variables in women was stronger than that in men. Figures 2 and 3 show a comparison of the predictive power of several variables based on the AUCs in men and women.

**Table 4:** Analysis of the predictive power of the individual measures using AUCs

Variables	Women	Men
Age	0.687 (0.652–0.722)	0.517 (0.485–0.548)
Height	0.614 (0.574–0.654)	0.539 (0.505–0.572)
Weight	0.622 (0.581–0.662)	0.606 (0.574–0.639)
WC	0.718 (0.685–0.751)	0.670 (0.641–0.700)
BMI	0.695 (0.661–0.730)	0.652 (0.621–0.683)
Wd-A	0.504 (0.466–0.543)	0.531 (0.496–0.565)
LRb-A	0.607 (0.565–0.649)	0.633 (0.601–0.665)
RRb-A	0.653 (0.614–0.691)	0.619 (0.586–0.651)
LS-A	0.541 (0.497–0.584)	0.505 (0.471–0.540)
Plv-A	0.696 (0.660–0.731)	0.596 (0.564–0.629)
Wd-BMC	0.609 (0.569–0.649)	0.492 (0.458–0.525)
LRb-BMC	0.564 (0.521–0.607)	0.605 (0.573–0.637)
RRb-BMC	0.609 (0.568–0.650)	0.601 (0.568–0.634)
LS-BMC	0.579 (0.537–0.622)	0.508 (0.474–0.541)
Plv-BMC	0.637 (0.596–0.678)	0.528 (0.494–0.561)
Wd-BMD	0.614 (0.573–0.655)	0.500 (0.467–0.534)
LRb-BMD	0.530 (0.487–0.573)	0.509 (0.476–0.542)
RRb-BMD	0.548 (0.504–0.592)	0.516 (0.483–0.549)
LS-BMD	0.602 (0.560–0.644)	0.495 (0.462–0.528)
Plv-BMD	0.508 (0.464–0.551)	0.560 (0.527–0.593)
LArm-Ft	0.629 (0.590–0.667)	0.639 (0.608–0.670)
RArm-Ft	0.638 (0.599–0.676)	0.626 (0.594–0.657)
Trk-Ft	0.715 (0.684–0.747)	0.672 (0.643–0.702)
WBT-Ft	0.650 (0.614–0.686)	0.647 (0.616–0.677)
LArm-Ln	0.553 (0.509–0.598)	0.529 (0.494–0.563)
RArm-Ln	0.569 (0.526–0.612)	0.521 (0.486–0.555)
Trk-Ln	0.569 (0.524–0.613)	0.559 (0.525–0.593)
WBT-Ln	0.552 (0.508–0.596)	0.545 (0.511–0.580)
LArm-Ms	0.618 (0.576–0.660)	0.582 (0.549–0.615)
RArm-Ms	0.631 (0.590–0.672)	0.574 (0.540–0.607)
Trk-Ms	0.678 (0.641–0.714)	0.637 (0.605–0.669)
WBT-Ms	0.621 (0.580–0.661)	0.606 (0.573–0.639)

*AUC* Area under the receiver operating characteristic curve. AUC values were calculated by creating ROC curves using SPSS. *WC* Waist circumferencem, *BMI* Body mass index, *Wd-A* Ward’s triangle area, *LRb-A* Left rib area, *RRb-A* Right rib area, *LS-A* Lumbar spine area, *Plv-A* Pelvis area, *Wd-BMC* Ward’s triangle bone mineral content, *LRb-BMC* Left rib bone mineral content, *RRb-BMC* Right rib bone mineral content, *LS-BMC* Lumbar spine bone mineral content, *Plv-BMC* Pelvis bone mineral content, *LRb-BMD* Ward’s triangle bone mineral density, *Wd-BMD* Left rib bone mineral density, *RRb-BMD* Right rib bone mineral density, *LS-BMD* Lumbar spine bone mineral density, *Plv-BMD* Pelvis bone mineral density, *LArm-Ft* Left arm fat mass, *RArm-Ft* Right arm fat mass, *Trk-Ft* Trunk fat mass, *WBT-Ft* Whole body total fat mass, *LArm-Ln* Left arm lean mass, *RArm-Ln* Right arm lean mass, *Trk-Ln* Trunk lean mass, *WBT-Ln* Whole body total lean mass, *LArm-Ms* Left arm mass, *RArm-Ms* Right arm mass, *Trk-Ms* Trunk mass, *WBT-Ms* Whole body total mass

## Discussion

High TG levels are clearly associated with various diseases, such as CVD [2–4], CHD [5–7], insulin resistance syndrome [12, 13], metabolic syndrome [8–11], and abdominal obesity [9, 12, 15–17].

Numerous studies have investigated the best indicators of hypertriglyceridemia. Hypertriglyceridemia or TG levels are associated with anthropometric measures. As shown in a study conducted by Ghosh et al. [20], WC and the WHR are significantly and positively correlated with TG levels in middle-aged Bengalee Hindu men. According to Sharp et al. [21], WC is the single best indicator of the risk of disease, including CVD, in Hispanic and Caucasian adolescents. In a study conducted by Lee et al. [22], WC exhibited the best predictive power for hypertriglyceridemia in Korean adults. Lee et al. [23] also reported that the WHtR was the best discriminator of dyslipidemia in both men and women. Moreover, women in whom dyslipidemia was identified tended to have higher AUCs than men, which is consistent with the results of the present study. Based on the results reported by Lee et al. [24], age is the highest risk factor in women, and the anthropometric measures of WHtR in women and the RFcR in men are the strongest indicators of hypertriglyceridemia. Most previous studies using anthropometric measures have revealed that the WC or WHtR is an important indicator of hypertriglyceridemia or CVD. Our findings are consistent with the results of some previous studies [20–22] indicating that WC is an important risk factor for hypertriglyceridemia in women.

Many studies have also revealed a strong correlation between BMD and TG levels. According to Muhlen et al. [28], men and women with metabolic syndrome exhibit higher total hip BMDs than subjects without metabolic syndrome in an age-adjusted analysis. Men with metabolic syndrome also exhibit higher femoral neck BMDs. Akira et al. [29] revealed a correlation between osteoporosis and hypertriglyceridemia in postmenopausal women. As reported in a study conducted by Lawlor et al. [30], TG levels are positively associated with BMD, BMC, and BA in male but not female adolescents. Son et al. [31] reported that TG levels are positively correlated with bone density T-values, and a significant positive correlation was observed in healthy Korean men after correcting for age and BMI. In a study conducted by Cui et al. [32], the TG levels were significantly and positively correlated with the BMD at the trochanter site in postmenopausal women, and premenopausal women with TG levels in the higher quartile exhibited lower lumbar BMD values. Dennison et al. [33] revealed correlations between BMDs measured at the lumbar spine and total femoral region and the serum TG levels. Yujie et al. [35] showed that the TG levels were directly correlated with the BMD at the lumbar spine in type 2 diabetes patients. Saoji et al. [36] reported that the BMD at the spine and femur site was associated with TG in women from Northeast India of Tibeto-Burman origin. Mirzababaei et al. [16] postulated that high serum TG levels and low serum HDL-C levels exerted mediating effects on the relationship between obesity and high BMD at the hip region in metabolically unhealthy obese subjects in Iran. Yoldemir et al. [34] reported weak negative correlations between TG levels and BMD at the lumbar spine in healthy postmenopausal Turkish women. However, the relationship between hypertriglyceridemia or high TG levels and BMD remains controversial. According to Yamaguchi et al. [37], plasma TG levels are not correlated with BMD values at any skeletal site. Kim et al. [39] failed to observe correlations between TG levels and BMDs measured at any site in postmenopausal Korean women. Sung et al. [38] reported that TG levels were not correlated with BMDs in elderly Korean men. Lilianne et al. [40] failed to observe significant correlations between TG levels and BMDs measured at various skeletal sites, such as the lumbar spine, femoral neck, and total hip. Li et al. [41] did not identify an association between TG levels and BMD in postmenopausal Chinese women. In the present study, the area and BMC of the pelvis in women and the area and BMC of the left rib in men were the most important indicators of hypertriglyceridemia. These results are similar to those reported in previous studies [16, 28–36], indicating that BMD is correlated with TG levels.

The TG levels are associated with the fat mass in the abdominal region. In a study conducted by Kissebah et al. [12], high plasma TG levels were correlated with upper body obesity. Despres et al. [26] reported that abdominal fat was associated with low serum HDL-C concentrations. Additionally, obesity and abdominal fat accumulation were associated with hypertriglyceridemia, and high plasma TG levels were associated with TGs enriched in LDL and HDL in another study [15]. As reported by Lipsky et al. [4], the trunk fat mass was positively correlated with the TG levels. According to a simple correlation analysis reported by Takeuchi et al. [14], the trunk/leg fat ratio is strongly and positively correlated with TG levels and postprandial triglyceridemia in young Japanese women, and leg and trunk fat are negatively and positively correlated, respectively, with TG levels and postprandial triglyceridemia after mutual adjustment. Lee et al. [27] showed that the TG level was significantly correlated with trunk fat in an obese group of patients with gastric neoplasms over 1 year of follow-up after laparoscopic gastrectomy. The present results are consistent with some previous studies [4, 14, 15, 26] and indicate strong associations between upper body fat mass, particularly trunk fat mass, and TG levels.

TG levels, bone mineral density, and body fat mass have been reported to differ among ethnic and race groups [14, 46–51]. Sharma et al. [46] reported lower TG concentrations in African-Americans than Whites or Hispanics diagnosed with type 2 diabetes mellitus. Marcus et al. [47] examined the correlations with BMD in a postmenopausal estrogen/progestin intervention trial and reported that black women exhibited the highest 2nd-4th lumbar spine BMD, and Hispanic women exhibited the highest femoral neck BMD. Araujo et al. [48] reported that the BMC and BMD values in black men were greater than those in Hispanic or white men. These authors proposed that the differences in BMC and BMD potentially explain the variations in the fracture rates among black, Hispanic, and white men. Lu et al. [49] reported that ethnicity exerted the strongest effect on most regional body BMD values among Chinese, white, and black subjects across both men and women. George et al. [50] reported that the whole body, hip, femoral neck and lumbar spine BMD values in black African subjects were significantly higher than those in Indian subjects in South Africa. Keswell et al. [13] described differences in body fat composition according to ethnicity. Based on their findings, black women are significantly shorter and heavier and present a higher BMI and greater fat mass than white women, and black women exhibit greater absolute trunk, leg and arm fat mass measurement values than white women. Moreover, black women exhibit lower TG concentrations and higher trunk fat masses than white women. Additionally, the associations between the body fat composition and TG levels differ by ethnicity and race [13, 25]. Hosain et al. [25] reported ethnicity- and race-specific differences in correlations between body fat distribution variables and serum lipid profiles, including TG levels, among reproductive-age black, white and Hispanic women. According to Keswell et al. [13], a higher trunk fat mass in black women and a higher visceral adipose tissue mass in white women are associated with TG concentrations.

Several studies have described gender differences in correlations between BMDs and TG levels and metabolic syndrome. As shown in a study conducted by Lawlor et al. [30], the TG concentrations are positively correlated with BMD, BMC, and BA in adolescent men but not in women. Kim et al. [10] indicated that the BMD is negatively correlated with the TG levels in men but not in women. In a study conducted by Muka et al. [52], WC was inversely correlated with the FNk-BMD in men, and the HDL-C concentrations were positively correlated with the FNk-BMD in women but not in men. Loke et al. [11] reported that metabolic syndrome was positively correlated with BMD in men and negatively correlated with BMD in women in Taiwanese elderly populations. In the present study, WC in women and Trk-Ft in men were the best indicators of hypertriglyceridemia. Our results support some previous studies identifying gender differences in correlations between TG levels and BMDs in metabolic syndrome patients [10, 11, 30, 52]. The present study has several limitations. First, cause-effect associations are difficult to determine because of the cross-sectional design. Second, our results were limited to Korean adults because we used data from the fifth Korea National Health and Nutrition Examination Survey in this study. Despite these limitations, the results of this and previous studies support that anthropometric indices, such as WC, WHtR, and WHR, are associated with TG levels. Therefore, anthropometric indices may be used for the identification of hypertriglyceridemia or TG levels in initial health screening efforts. However, although the BMD was associated with the TG levels in our results, this association remains controversial because there are conflicting arguments in many studies.

## Conclusion

The present study examined anthropometric variables, bone density and body fat composition (bone area, BMC, BMD, body fat mass, and lean body mass) in Korean adults and showed that WC in women and Trk-Ft in men exhibited the best predictive power for hypertriglyceridemia. WC and Trk-Ft exhibited similar predictive powers for hypertriglyceridemia in both women and men. Moreover, WC and Trk-Ft exhibited greater predictive power in women than in men. Our findings provide clinical information that may be useful for the identification of hypertriglyceridemia or high TG levels during initial screening steps. Further studies are needed to build a model for accurate identification based on a combination of BMD, anthropometric, and fat mass data.

## Abbreviations

AUC: Area under the receiver operating characteristic curve
BMC: Bone mineral content
BMD: Bone mineral density
DXA: Dual energy X-ray absorptiometry
HDL-C: High density lipoprotein cholesterol
LDL-C: Low density lipoprotein cholesterol
TC: Total cholesterol
TG: Triglyceride
Trk-Ft: Trunk fat mass
WC: Waist circumference
WHR: Waist-to-hip ratio
WHtR: Waist-to-height ratio
